# Comprehensive Gene-Based Association Study of a Chromosome 20 Linked Region Implicates Novel Risk Loci for Depressive Symptoms in Psychotic Illness

**DOI:** 10.1371/journal.pone.0021440

**Published:** 2011-12-29

**Authors:** T. Bernard Bigdeli, Brion S. Maher, Zhongming Zhao, Edwin J. C. G. van den Oord, Dawn L. Thiselton, Jingchun Sun, Bradley T. Webb, Richard L. Amdur, Brandon Wormley, Francis A. O'Neill, Dermot Walsh, Brien P. Riley, Kenneth S. Kendler, Ayman H. Fanous

**Affiliations:** 1 Department of Human and Molecular Genetics, Virginia Commonwealth University, Richmond, Virginia, United States of America; 2 Virginia Institute for Psychiatric and Behavioral Genetics, Virginia Commonwealth University, Richmond, Virginia, United States of America; 3 Department of Psychiatry, Virginia Commonwealth University, Richmond, Virginia, United States of America; 4 Department of Mental Health, Johns Hopkins Bloomberg School of Public Health, Baltimore, Maryland, United States of America; 5 Departments of Psychiatry, Biomedical Informatics, and Cancer Biology, Vanderbilt University Medical Center, Vanderbilt, Tennessee, United States of America; 6 Center for Biomarker Research and Personalized Medicine, Virginia Commonwealth University, Richmond, Virginia, United States of America; 7 Mental Health Service Line, Washington VA Medical Center, Washington, D. C., United States of America; 8 Department of Psychiatry, Keck School of Medicine of the University of Southern California, Los Angeles, California, United States of America; 9 Department of Psychiatry, Queens University, Belfast, United Kingdom; 10 The Health Research Board, Dublin, Ireland; 11 Department of Psychiatry, Georgetown University School of Medicine, Washington, D. C., United States of America; Baylor College of Medicine, United States of America

## Abstract

**Background:**

Prior genomewide scans of schizophrenia support evidence of linkage to regions of chromosome 20. However, association analyses have yet to provide support for any etiologically relevant variants.

**Methods:**

We analyzed 2988 LD-tagging single nucleotide polymorphisms (SNPs) in 327 genes on chromosome 20, to test for association with schizophrenia in 270 Irish high-density families (ISHDSF, N = 270 families, 1408 subjects). These SNPs were genotyped using an Illumina iSelect genotyping array which employs the Infinium assay. Given a previous report of novel linkage with chromosome 20p using latent classes of psychotic illness in this sample, association analysis was also conducted for each of five factor-derived scores based on the Operational Criteria Checklist for Psychotic Illness (delusions, hallucinations, mania, depression, and negative symptoms). Tests of association were conducted using the PDTPHASE and QPDTPHASE packages of UNPHASED. Empirical estimates of gene-wise significance were obtained by adaptive permutation of a) the smallest observed *P-*value and b) the threshold-truncated product of *P-*values for each locus.

**Results:**

While no single variant was significant after LD-corrected Bonferroni-correction, our gene-dropping analyses identified loci which exceeded empirical significance criteria for both gene-based tests. Namely, *R3HDML* and *C20orf39* are significantly associated with depressive symptoms of schizophrenia (*P*_emp_<2×10^−5^) based on the minimum *P-*value and truncated-product methods, respectively.

**Conclusions:**

Using a gene-based approach to family-based association, *R3HDML* and *C20orf39* were found to be significantly associated with clinical dimensions of schizophrenia. These findings demonstrate the efficacy of gene-based analysis and support previous evidence that chromosome 20 may harbor schizophrenia susceptibility or modifier loci.

## Introduction

With a lifetime prevalence of 1 percent and an estimated annual cost of $62.7 billion in the United States [1], schizophrenia (Scz) is a debilitating neuropsychiatric disorder which poses a significant burden to public health. Whether schizophrenia represents a single or multiple disease processes is a source of persistent controversy, as patients vary considerably in onset, course and outcome of disease, and the particular combination of symptoms endorsed [2], [3]. Models comprising continuous traits—often extracted in factor analysis of symptom profiles—have been adduced, typically distinguishing positive, negative, disorganization, and affective symptoms [4]. One explanation for this variability lies in the existence of more than one putative etiopathogenic mechanism, each imparting susceptibility to a more or less distinct disease subtype or influencing the character of illness dimensionally. Detection and subsequent replication of several putative risk variants, facilitated by genome-wide association studies (GWAS) [5]–[10], has seen renewed interest in this question among geneticists and diagnosticians alike [11]–[13].

Consistent with the observed variability in clinical presentation is the hypothesis that schizophrenia is likely genetically heterogeneous [6], [8]. Linkage and candidate gene association studies have implicated a number of genes and genomic regions, with varying degrees of subsequent independent replication. Allelic heterogeneity has been demonstrated in meta-analyses of candidate genes such as *DTNBP1* [14], [15]. If the observed clinical heterogeneity of schizophrenia is in fact due to genetic heterogeneity, the use of more clinically homogenous phenotypes may increase the signal-to-noise ratio in gene-finding studies. A previous report by our group described detection of novel linkage to 20p using latent classes of psychotic illness [16]. Linkage analysis of Mania, Schizomania, Deficit Syndrome and Core Schizophrenia latent classes yielded several suggestively significant loci, in regions of chromosome 20 which had previously yielded very little evidence of linkage in our sample. Furthermore, the presence of susceptibility genes in chromosome 20 has been suggested by several previous linkage studies as well [17]–[23]. In addition to genes which increase susceptibility to more or less distinct clinical subtypes of illness, other genes may influence clinical features of disease in a dimensional fashion, without altering liability to the illness itself. These have previously been described as modifier loci [2]. Modifier loci may not be resolvable using traditional dichotomous phenotypes (simply “affected” or “unaffected”), but rather, by quantitative symptomatic measures. Several examples have been reported [24]–[30].

Recent GWAS of schizophrenia support a polygenic model in which potentially thousands of common variants individually impart small effects. Given the unprecedented multiple-comparison burden incurred in a genome-wide approach, hypothesis-based strategies remain viable alternatives for the study of complex disease. A gene-based approach is particularly convenient. In an analysis of bipolar and schizophrenia datasets, Moskvina and colleagues [31] observed significantly more SNPs within genes showing evidence for association than expected, with intergenic SNPs showing no such trend.

We describe a comprehensive, gene-based association survey of 327 genes in regions linked to chromosome 20 in our previous studies. In addition to testing for association with traditional diagnostic definitions of schizophrenia, we also sought to assess whether chromosome 20 harbors modifier loci. Association analysis was therefore also performed for five factor-derived scores, representing hallucinations, delusions, depressive symptoms, manic symptoms, and negative symptoms, in schizophrenia cases only. In addition to single-marker tests of allelic association, we employ two gene-based test-statistics, the minimum observed *P-*value per gene and the truncated product of *P-*values, to evaluate the efficacy of a gene-based approach as applied to a large, family-based study.

## Results

### Gene-wide Association Analyses

**Figure 1:**
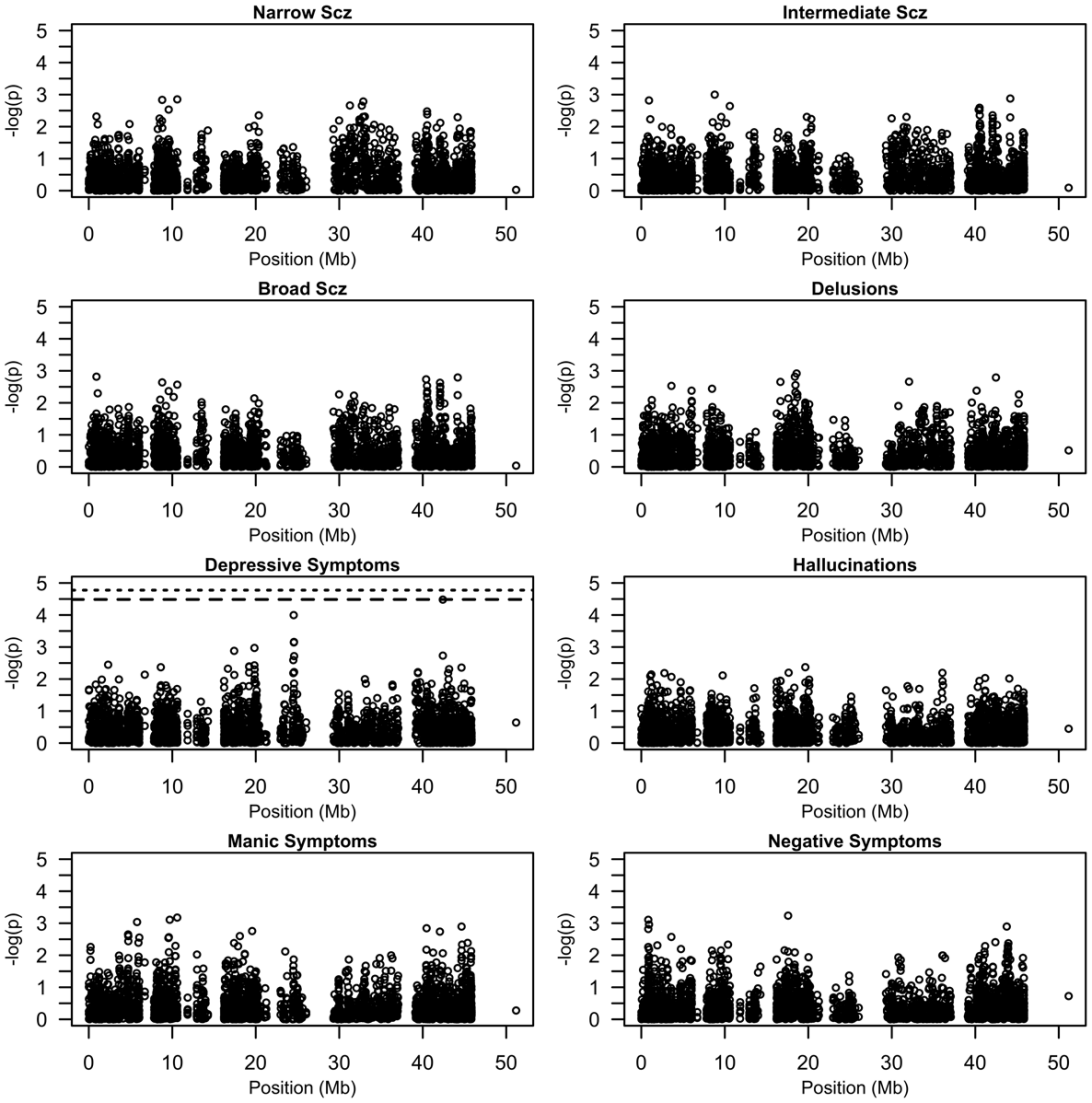
Physical distribution of single-marker associations on chromosome 20, for both categorical diagnoses and clinical dimensions of Scz. Associations are displayed as log-transformed *P*-values (−*log*_10_ *P*) at genomic positions in megabases (Mb). Where appropriate, a dotted line indicates the Bonferroni-corrected significance threshold, accounting for number of SNPs assayed experiment-wide. Similarly, a dashed line indicates the LD-corrected significance threshold, as estimated by SNPSpD.

Following quality-control protocols, 2,988 single nucleotide polymorphisms in 327 genes were tested for association with a diagnosis of schizophrenia (Figure 1). Estimates of empiric significance (*P*_emp_) were obtained via an adaptive permutation procedure employing the smallest observed *P-*value, as well as the truncated product of *P-*values (α_trunc_≤0.01) per gene. The number of genes carried forward in successive stages of this procedure, in both approaches, can be found in Table 1. Using the minimum gene-wide *P-*value approach, no genes were observed to be significantly associated with narrow (N = 1574), intermediate (N = 1749), or broad (N = 1808) diagnoses of schizophrenia. Next, we sought to identify those SNPs associated with clinical dimensions of schizophrenia in a subset of cases (N = 721) for which the OPCRIT was available. A previous report by Fanous and colleagues [16] supports linkage of latent classes derived from the OPCRIT to chromosome 20 in this sample. No genes were found to be significantly associated with the negative, manic, hallucinations or delusions factors. In the analysis of the clinical dimensions, *R3HDML* demonstrated significant evidence of association (*P*_emp_<2×10^−5^) with the depressive factor using the minimum *P-*value approach. Using the truncated product of *P-*values, *C20orf39* was also found to be significantly associated with the depressive factor (*P*_emp_<2×10−5). It is important to note that, for both *C20orf39* and *R3HDML*, we observed fewer than ten simulated results more significant than the observed test-statistic after 100,000 permutations. Hence, our estimates of empirical significance may be conservative. However, extending our analyses to 1 *M* permutations was not carried out as it was too computationally demanding.

**Table 1:** Number of genes requiring additional simulations at each stage of adaptive permutation.

	Trait	Min *P* (100)	Min *P* (1 K)	Min *P* (10 K)	Min *P* (100 K)	Trunc Prod *P* (100)	Trunc Prod *P* (1 K)	Trunc Prod *P* (10 K)	Trunc Prod *P* (100 K)
*Diagnostic Category*	Narrow	19	5	0	0	9	5	0	0
*Diagnostic Category*	Int	12	4	0	0	6	4	2	0
*Diagnostic Category*	Broad	14	3	0	0	5	3	0	0
*Symptom Factor*	del	15	4	0	0	8	4	0	0
*Symptom Factor*	dep	10	6	1	1†	9	4	2	1‡
*Symptom Factor*	hal	6	2	0	0	4	1	0	0
*Symptom Factor*	manic	5	2	0	0	14	6	0	0
*Symptom Factor*	neg	20	4	0	0	14	7	0	0

For each diagnosis and symptom factor, the number of loci requiring additional permutations after each stage of our adaptive procedure, given for both the Min *P* and truncated-product methods. For an observed test-statistic to be considered significant at a particular stage of permutation, there may be no greater than 10 simulated null statistics which are more extreme than the observed. For each gene-based test, the number of permutations performed at each stage is displayed parenthetically (100, 1,000, 10,000, and 100,000). *Dim* codes “del”, “dep”, “hal”, “manic”, “neg” are delusions, depressive symptoms, hallucinations, mania, and negative symptoms, respectively, and described elsewhere in full.

† Min P finding for R3HDML.

‡ truncated-product finding for C20orf39.

Because validation of a truncated product approach in extended pedigrees relies on the permutation procedure faithfully conserving patterns of LD within each replicate dataset, we obtained a quantitative measure of how well haplotype-block structure was maintained for *C20orf39* across actual and simulated datasets. In calculating an LD-corrected significance threshold, SNPSpD estimates the effective number of independent tests present in a set of markers. Using SNPSpD, 1,000 replicate datasets for *C20orf39* were assessed for number of independent tests. When compared to the estimate based on the actual pattern of LD in *C20orf39* (i.e., 26 independent tests), the distribution of these simulation-derived estimates demonstrates that the LD structure within each replicate does not differ significantly from the observed data (*P*≈0.409; 95% CI: [26], [28]). This increases confidence in the truncated product finding for *C20orf39*. However, this may not hold for every gene and may be sensitive to specific patterns of linkage disequilibrium.

### Single Marker Association Analysis

Taking each SNP to represent an independent hypothesis but correcting for LD using SNPsPD, we found that no single marker met experiment-wide criteria for association (α_SNPsPD_<3.18×10−5) with either the three categorical diagnostic definitions used or our OPCRIT-derived factor scores (Tables 2, 3). The strongest evidence of association with a diagnosis of schizophrenia was in *PLCB1* (20p12.3) (rs6108205, *P*≈1.00×10^−3^, intermediate Scz diagnosis). For the depressive factor, we observed the strongest associations experiment-wide at 20q13.12 (rs3761184, *P*≈3.31×10^−5^) in *R3HDML* This was very close to the LD-corrected significance threshold calculated using SNPSpD (*P* = 3.18×10^−5^). Furthermore, rs11700002, in *C20orf39* at 20p11.21 attained *P*≈1.01×10^−4^.

**Table 2:** Top ten Pedigree Disequilibrium Test results for categorical diagnoses of Schizophrenia.

Chr/Mb	Gene	dbSNP	Nuc.(Minor)	Assoc.	*Frq_assoc_*	Trios(Tr/NTr)	DSPs(Aff/Unaff)	*Z*-score	*x^2^*	*P*	Dx
20/0.92	*RSPO4*	rs6056462	A/G(G)	A	0.842	94/90	868/855	3.171	10.05	1.52×10^−3^	Int
20/0.92	*RSPO4*	rs6056462	A/G(G)	A	0.843	97/93	911/897	3.170	10.05	1.53×10^−3^	Broad
20/8.79	*PLCB1*	rs6108205	C/T(C)	C	0.491	67/65	536/467	3.289	10.82	1.00×10^−3^	Int
20/8.79	*PLCB1*	rs6108205	C/T(C)	C	0.490	58/56	444/391	3.182	10.13	1.46×10^−3^	Narrow
20/10.60	*JAG1*	rs6133987	C/T(T)	C	0.778	85/78	678/610	3.193	10.19	1.41×10^−3^	Narrow
20/40.41	*PTPRT*	rs6072690	A/G(A)	A	0.442	67/59	586/502	3.224	10.40	1.26×10^−3^	Broad
20/40.51	*PTPRT*	rs6130134	C/T(T)	C	0.792	94/93	880/826	3.152	9.940	1.62×10^−3^	Int
20/42.07	*TOX2*	rs6103560	C/T(T)	C	0.705	111/95	855/822	3.200	10.24	1.37×10^−3^	Broad
20/44.19	*CD40*	rs3765457	A/G(G)	G	0.175	24/24	235/170	3.154	9.950	1.33×10^−3^	Broad
20/44.19	*CD40*	rs3765457	A/G(G)	G	0.176	23/23	223/160	3.210	10.30	1.61×10^−3^	Int

For each gene, *Chr/Mb* denotes chromosome and genomic position (Megabases), *dbSNP* is the rs-identifier for the assayed SNP, and *Nuc* is the nucleotide substitution at a SNP. *Frq_assoc_* and *Z*-scores are with respect to the associated allele. Allelic transmissions from parent to affected child is given by *Trios*, where *Tr* and *NTr* represent number of transmissions and non-transmissions. Allele-sharing between phenotypically-discordant sib-pairs is given by *DSPs*, with *Aff/Unaff* denoting the number of associated alleles in affected and unaffected siblings, respectively. *Dx* codes “Core”, “Int”, and “Broad” are Core, Intermediate, and Broad diagnoses of schizophrenia, respectively, and are described elsewhere in full.

**Table 3:** Top ten Quantitative Pedigree Disequilibrium Test results for clinical dimensions of Schizophrenia.

Chr/Mb	Gene	dbSNP	Nuc.(Minor)	Assoc.	Frq*_assoc_*	Gametes (maj/min)	*Z*-score	*x^2^*	*P*	*Dim*
20/0.83	*ANGPT4*	rs976166	C/G(C)	C	0.308	1285/571	3.333	11.11	8.59×10^−4^	neg
20/9.70	*PAK7*	rs2327225	A/C(C)	A	0.718	1333/523	3.426	11.74	6.13×10^−4^	manic
20/10.59	*JAG1*	rs6133986	A/G(A)	A	0.089	1691/165	3.523	12.41	4.27×10^−4^	manic
20/16.66	*SNRPB2*	rs6111262	C/T(T)	C	0.720	1337/519	3.281	10.76	1.04×10^−3^	del
20/17.58	*RRBP1*	rs3790310	A/T(A)	T	0.809	1502/354	3.451	11.91	5.58×10^−4^	neg
20/24.54	*C20orf39*	rs11700002	A/G(A)	A	0.187	1509/347	3.930	15.44	8.50×10^−5^	dep
20/24.56	*C20orf39*	rs4815292	G/T(G)	G	0.353	1201/655	3.362	11.30	7.73×10^−4^	dep
20/24.58	*C20orf39*	rs11696125	G/T(T)	T	0.230	1430/426	3.392	11.50	6.94×10^−4^	dep
20/24.58	*C20orf39*	rs11087473	A/G(A)	A	0.230	1430/426	3.392	11.50	6.94×10^−4^	dep
20/42.40	*R3HDML*	rs3761184	A/G(G)	G	0.169	1543/313	4.120	16.98	3.78×10^−5^	dep

For each gene, *Chr/Mb* denotes chromosome and genomic position (Megabases), *dbSNP* is the rs-identifier for the assayed SNP, and *Nuc* is the nucleotide substitution at a SNP. *Gametes* represents the number of major and minor alleles (*maj/min*) transmitted from parent to affected child or unshared between phenotypically-discordant sib-pairs. *Frq_assoc_* and *Z*-scores are with respect to the allele corresponding to a higher trait mean. *Dim* codes “del”, “dep”, “hal”, “manic”, “neg” are delusions, depressive symptoms, hallucinations, mania, and negative symptoms, respectively, and described elsewhere in full.

## Discussion

We have conducted a comprehensive gene-based association study of 327 genes on chromosome 20 in an Irish sample of 270 high-density schizophrenia families. This study sought to identify common variants conferring susceptibility to schizophrenia, following up reported linkage in this sample to clinical subtypes of psychotic illness [16], as well as previous studies reporting linkage to chromosome 20. Because those clinical subtypes were derived from quantitative symptom dimensions, we also tested for association with these same dimensions. Although traditional single-marker tests failed to identify any SNPs meeting experiment-wide criteria for significance, application of gene-wide association metrics revealed two previously unimplicated loci, *R3HDML* and *C20orf39*, associated with depressive symptoms. Our findings support the power of gene-based association approaches. They also lend further support to previous evidence suggesting that genetic differences may underlie clinical heterogeneity in schizophrenia [2], [3].

**Figure 2:**
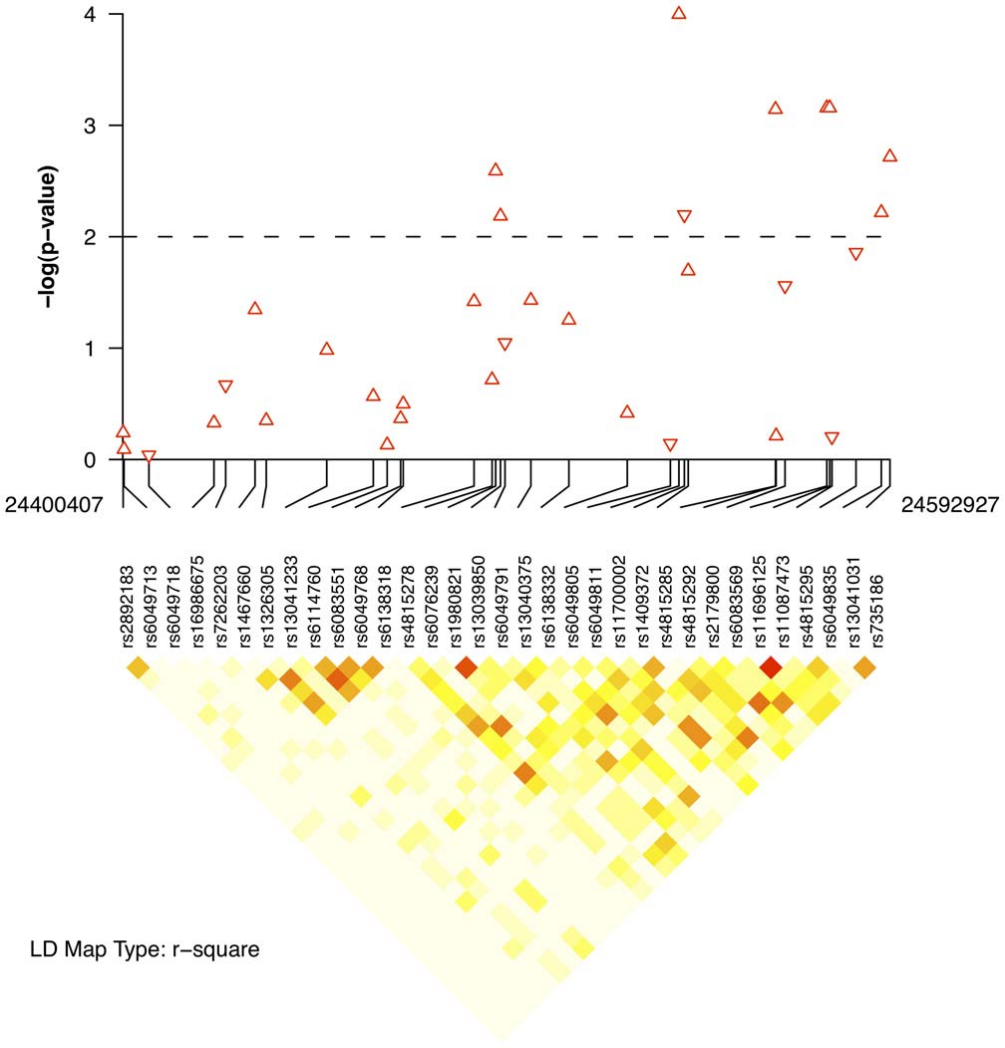
Association of *C20orf39* SNPs with depressive symptoms of Scz. Magnitudes and directions of associations are displayed in the upper panel, with upwards-oriented triangles indicating a positive correlation with symptom factor score. A dashed line is provided at the inclusion threshold for the truncated product of *P*-values. Connecting lines relate the physical positions of associations to SNP labels in the corresponding LD-map (*r* ^2^). Plot generated using snp.plotter for R [66].

One of the aims of this study was to identify genomic loci predisposing to a particular form of illness or which modifies clinical presentation amongst affected individuals. Such genes have been described previously as “modifier” or “susceptibility-modifier” loci and are reviewed elsewhere [2]. Of the two loci showing the strongest associations, namely *R3HDML* and *C20orf39*, neither appears to affect the risk of the illness itself. That is, no single variant in either gene met even nominal significance criteria (*P*<0.05) for association with narrow, intermediate, or broad diagnoses of schizophrenia. These two genes would therefore fulfill our definition of modifier genes [2]. However, the strength of evidence we observed for *R3HDML* is greater than that observed for *C20orf39*. *R3HDML* was identified by application of the minimum *P-*value approach. Among affected individuals, those carrying the minor allele (G) of the corresponding SNP, rs3761184, had higher mean depression scores. On the other hand, for *C20orf39*, empirical significance was attained using the truncated product of *P-*values. This makes it more difficult to identify a specific risk genotype. This is because the truncated product method only considers all variation within a gene jointly. In Figure 2, it is apparent that those markers contributing to the truncated product for *C20orf39* comprise a block of LD distinct from the surrounding region, with the majority showing association of the minor allele with higher depression scores. Whereas individually, none of the single-marker associations were significant after our permutation procedure, the degree of correlation between the SNPs may have been sufficient to produce an empirically significant association for *C20orf39* as a whole. In order to rule out a spurious gene-wise association due to higher LD, we analyzed a set of permutations using SNPSpD, then compared the distribution of estimated number of independent tests (SNPs) to that obtained for the actual data. If our gene-dropping simulations were found to consistently underestimate the extent of LD between adjacent markers—indicated by a larger number of independent tests—we would expect an inflation of the empiric test-statistic. Alternatively, if the observed LD within simulated datasets tended to overestimate pairwise LD, the corresponding distribution of truncated products would underestimate the empiric test-statistic. For *C20orf39*, the observed SNPSpD estimate of ∼ 26 tests was not found to differ significantly from the null distribution of simulated datasets, suggesting that our gene-dropping procedure was faithfully conserving LD-structure across our simulations. As discussed, increased gene-size, especially in the presence of higher LD between markers, might also contribute to over-estimation of the test statistic.

To our knowledge, neither *R3HDML* nor *C20orf39* has been functionally characterized to date. Both are predicted genes identified on the basis of domain homology. The *R3HDML* locus encodes a putative serine protease inhibitor belonging to the CRISP family of cysteine-rich secretory proteins, and contains evolutionarily conserved exonic and intronic regions bearing greater than 90% similarity to Rhesus macaque [32]. Interspersed within the conserved intronic sequences are numerous stretches of simple tandem repeats (e.g. CG_n_). Our SNP of interest in *R3HDML*, rs3761184, falls just upstream (<50 bp) of the second exon and 150 bp downstream of one such repeat-rich region. Roles in fertilization, spermatogenesis, and pathogen response have all been proposed for CRISP proteins, but these mechanisms are not immediately supportive of *R3HDML* as a schizophrenia candidate gene. However, recent implication of a number of HLA genes in large-scale GWAS suggest that genes involved in immune-related mechanisms, such as pathogen response, could be reasonable Scz candidates [8]. The presence of specific sequence features in the vicinity of the associated SNP may warrant more thorough bioinformatic inquiry. Additionally, *R3HDML* lies approximately 57 kb downstream of the *GDAP1L1* locus, which appears to encode a gluthionine S-transferase (GST). Cell-culture studies have demonstrated a relationship between gluthionine deficiency and oxidative stress, mechanisms frequently purported to contribute to schizophrenia pathophysiology [33], [34]. However, *GDAP1L1* was not significantly associated.

Our empirically significant finding for *C20orf39* presents additional challenges for interpretation, given its provisional status as an “open reading frame”. Provisionally known as *TMEM90B*, this locus encodes a predicted transmembrane protein. Of 33 SNPs assayed within *C20orf39*, the nine included in the truncated product bounded a region of LD corresponding to the coding region of *C20orf39*. The upstream, untranslated region of *C20orf39*, which itself corresponds to a distinct set of ESTs, yielded no SNPs meeting local significance criteria. Whether the markers driving this association simply lie in joint linkage disequilibrium with nearby causal variation, or actually demarcate an etiologically relevant genomic region, is unknown.

Depressive symptoms, especially suicidal ideation, comprise a considerable portion of morbidity and mortality in schizophrenia [35]. Therefore, follow up of these two genes could be important in the search for clues to more successful identification and treatment of this clinical dimension.

As demonstrated by Moskvina *et al.*, polymorphisms mapping to functional elements are more likely to be associated with complex disease than intergenic variation [31]. Despite ongoing annotation and characterization of functional elements, however, our knowledge of genomic variation, functional or otherwise, remains incomplete. This is exemplified by *C20orf39* and *R3HDML*, which are novel and unannotated.

A major benefit of gene-based approaches is that they are robust to allelic and haplotypic heterogeneity across samples. This makes them particularly suited for use in replication and meta-analysis. In traditional replication of single-marker associations, the associated SNP in the discovery sample is usually assayed in all subsequent replication samples. This could inflate Type-II error in the presence of population differences in haplotype structure and allele frequencies [36]. Complex patterns of associations, whether spurious or due to genetic heterogeneity, have been more the rule rather than the exception in candidate gene studies of complex disease, as demonstrated by studies of *DTNBP1* [14], [15]. For discovery-based approaches, adoption of a gene-based strategy may be of even more immediate benefit, specifically by providing a straightforward means of multiple-test correction. Furthermore, traditional methods to correct for multiple-testing, such as Bonferroni correction or the less overtly conservative SNPSpD method, may be less robust in detecting small genetic effects. However, in spite of the advantages of gene-based association studies intergenic causative variants or variants in unrecognized genes might have been missed in this study.

Given the poor spatial resolution of linkage and intrinsic differences between these methodologies, we are currently unable to fully relate our association findings with the results of our previously published linkage study of latent classes. However, it is notable that *R3HDML* is located in a region which was linked to the “deficit syndrome” latent class, for which members were substantially more likely to fall below the median for depressive symptoms. Despite failing to demonstrate any evidence of association with a diagnosis of schizophrenia, *R3HDML* may be associated with a disease subtype characterized by low levels of depression. Because subtyping precludes use of our full sample for association analysis, statistical power is insufficient to test this hypothesis. Other methods aiming to identify more clinically homogenous subgroups have been applied to linkage analysis of schizophrenia. In a study of 168 affected sibling pairs, Hamshere and colleagues [37] demonstrated that inclusion of major depression as a covariate yielded suggestive evidence of linkage at 20q11.21, while schizophrenia as a whole did not. Taken together, these studies are compelling in their support of 20q11 harboring genes relevant to the affective component of schizophrenia. Emerging evidence supports a role for genetic variants conferring risk of both schizophrenia and bipolar disorder [8], [38]. Furthermore, genome scans of both disorders have consistently implicated regions of chromosome 20 [39]–[44]. A recent study of 383 bipolar or schizoaffective relative pairs found suggestive linkage at 20q13.31 when conditioning on the presence of mood-incongruent psychosis, furthering the argument that chromosome 20 loci may have relevance to conditions containing admixtures of mood and psychotic symptoms [45].

The findings presented here provide additional support to published findings suggesting that schizophrenia modifier loci may exist on chromosome 20 and, more generally, that genetic differences underlie clinical heterogeneity in schizophrenia [46]. We await replication of the observed associations between these loci and either categorically defined illness or more or less distinct subtypes or clinical dimensions. There are two main limitations relevant to this study. First, the truncated product of *P-*values is particularly sensitive to patterns of LD (unpublished results), since markers could be significant only due to their LD with other significant markers. Applied to family-based analysis of extended pedigrees, the validity of gene-based testing relies on the permutation method realistically maintaining LD across simulated datasets. As discussed, for *C20orf39*, the LD structure for a random sample of simulated datasets did not differ significantly from the actual data (*P*>0.05). Second, our analysis of multiple symptom dimensions may increase the Type-I error rate due to multiple testing. However, as we have previously shown, these dimensions are correlated [47], making Bonferroni correction overly conservative. It remains unclear whether the failure of traditional approaches to detect experiment-wide significant loci reflects the spurious nature of these findings or simply the limited power of this sample. Ultimately, the genotype-phenotype correlations reported herein require confirmation in independent samples for which comparable symptom measures are available. We are unaware of other family-based schizophrenia samples in which OPCRIT data are readily available. However, this is likely to be attempted in case-control samples by the Psychiatric GWAS Consortium Cross-Disorders Group [13].

## Methods

### Ethics Statement

This research was approved by the Institutional Review Boards of Virginia Commonwealth University School of Medicine and the Washington VA Medical Center. All subjects gave verbal assent to participate in research, as this was the norm in Ireland at the time these data were collected.

### Sample

Fieldwork for the Irish Study of High Density Schizophrenia Families (ISHDSF) was conducted between April 1987 and November 1992, with probands ascertained from public psychiatric hospitals in Ireland and Northern Ireland [48]. Selection criteria were two or more first-degree relatives meeting DSM-III-R criteria for schizophrenia or poor-outcome schizoaffective disorder (PO-SAD). Diagnoses were based on the Structured Interview for DSM-III-R Diagnosis (SCID) [49]. Independent review of all pertinent diagnostic information was made blind to pedigree assignment and marker genotypes by KSK and DW, with each diagnostician making up to three best-estimate DSM-III-R diagnoses. The Operational Criteria Checklist for Psychotic Illness (OPCRIT) [50] was completed by KSK for all subjects with probable lifetime histories of hallucinations or delusions (N = 755; N = 722 genotyped). Our diagnostic schema contains 4 concentric definitions of affection: narrow (D2) (schizophrenia, PO-SAD, and simple schizophrenia) (N = 577), intermediate (D5) which adds to D2 schizotypal personality disorder, schizophreniform and delusional disorders, atypical psychosis and good-outcome SAD (N = 700), broad (D8) (all disorders which significantly aggregated in relatives of probands) (N = 754) and very broad (D9), including any psychiatric illness (N = 961). Exploratory and confirmatory factor analysis of the OPCRIT was conducted previously by Fanous *et al.* [47]. This yielded a five-factor solution, comprising depressive, manic, and negative symptoms, delusions and hallucinations. Factor-derived scores were obtained by summing the scores of all items belonging to each factor.

### Bioninformatics and SNP-selection

Using WebGestalt [51], a total of 378 genes were initially identified as mapping to the region of chromosome 20 corresponding to the peak NPL and to the positions corresponding to a NPL of at least 1 on either side, based on the Illumina version 4.0 linkage SNP map used for genotyping in a multicenter linkage study funded by R01-MH068881 [52]. While there was very little evidence of linkage in our published microsatellite-based scan [53], we did observe modest evidence using the map in the Holmans *et al*. study [52], which included our study sample (results available on request). We included predicted genes and open reading frames (ORFs) from the p-terminal to 45.85 Mb (20q13.13). Physical map positions for 362 genes were obtained from the UCSC Genome Browser (hg17/NCBI Build 35) [54]. Tagging SNPs were selected for each identified genomic region (excluding upstream and downstream regions of genes) using Tagger (r^2^≥0.8, minor allele frequency (MAF)≥0.1) [55], as applied to the HapMap CEPH dataset [56]. Of these, 31 genes were excluded on the basis of tagging SNPs being unavailable. After removing multiple occurrences of markers resulting from overlap of adjacent genomic regions, 3,386 SNPs in 331 genes were selected for inclusion (Table S1).

### Genotyping

Genotyping was conducted by Illumina, Inc. using a custom iSelect array, which employs the Infinium assay. In total, DNA for 1,128 individuals was submitted for genotyping of 3,386 SNPs. As SNP markers from several ongoing experiments were included on the same array, per-individual summary statistics reflect genotyping across a total of 7,500 SNPs. Average genotyping completion rate across all SNPs was 99.97%. Of 1,128 samples, 21 failed to yield usable genotypes. Genotypes were examined for apparent Mendelian incompatibilities using PEDCHECK v 1.1 [57] and removed for entire families where appropriate.

### Association Analysis

We performed association analysis for categorical diagnoses of schizophrenia using PDTPHASE (UNPHASED v. 2.404), an implementation of the pedigree disequilibrium test (PDT) with extensions to deal with uncertain haplotypes and missing data [58], [59]. The PDT is an extension of the transmission disequilibrium test (TDT) to examine general pedigree structures and is similarly a test of association in the presence of linkage. Association with quantitative measures of disease was assessed using QPDTPHASE (UNPHASED v. 2.404), an implementation of the quantitative trait PDT with extensions to deal with uncertain haplotypes and missing data [59], [60]. An LD-corrected significance threshold was obtained using the SNPSpD package for R [61], [62]. For 2,988 SNPs, SNPSpD calculated an estimated 1,569 independent tests, with a corresponding significance threshold of α_SNPSpD_≈3.18×10^−5^, maintaining the type I error rate at 5%.

### Gene-wide Tests of Empirical Significance

Estimates of empirical significance for association results were obtained by adaptive permutation of gene-dropping simulations created with MERLIN [63]. Simulated genotypes were of identical frequency, marker spacing, and pattern of missing data as the actual genotypes, with individual phenotypes and pedigree structure also preserved within each simulated dataset. For markers in linkage disequilibrium (r^2^≥0.1), alleles were simulated using the haplotype frequencies for the marker clusters. To reduce computation time, those pedigrees of complexity greater than 70 bits were omitted from calculation of allele and haplotype frequencies. Each simulated dataset was analyzed as described above in two ways: retaining the minimum *P-*value per gene, as well as the calculating the threshold-truncated product of *P-*values (α_trunc_≤0.01) per gene. For the set of single-SNP hypotheses corresponding to a gene, the truncated product method considers the product of only those *P-*values falling below a specified threshold, evaluating the probability of observing as significant a product by chance. Whereas Fisher's Combined Test assesses the overall evidence for departure from the null, the truncated product approach can be used to assess whether suggestive or significant findings are truly significant [64]. Previous reports support the use of a truncated product approach in conjunction with the PDT [65]. Empirical significance was calculated from the proportion of simulated gene-wise test statistics more significant than the actual results (r_obs_+1/n_perm_+1). We used an adaptive permutation procedure, by which empirical *P-*values were obtained for 100, 1,000, 10,000, and 100,000 simulations. Only those observed associations for which there were not at least ten more significant simulated results were carried forward to each successive stage of permutation analysis.

## Supporting Information

Table S1 **Chromosome 20 genes assayed, with corresponding boundary SNPs.** For each gene assayed, the corresponding number of SNPs, position of the first SNP^‡^ and its dbSNP identifier, and the position of the last SNP^‡^ and dbSNP identifier are given. *^‡^Where applicable i.e. for loci with available tag SNPs.*
(PDF)
