# Supplementary material for: Comprehensive Gene-Based Association Study of a Chromosome 20 Linked Region Implicates Novel Risk Loci for Depressive Symptoms in Psychotic Illness
Source: PLoS One. 2011 Dec 29;6(12):e21440. doi: 10.1371/journal.pone.0021440 (PMC3248394; doi:10.1371/journal.pone.0021440)
Supplement: Table S1 — Chromosome 20 genes assayed, with corresponding boundary SNPs. For each gene assayed, the corresponding number of SNPs, position of the first SNP‡ and its dbSNP identifier, and the position of the last SNP‡ and dbSNP identifier are given. ‡Where applicable i.e. for loci with available tag SNPs. (PDF) [file pone.0021440.s001.pdf]

Table S1. Chromosome 20 genes assayed, with corresponding boundary SNPs.

Locus	No. SNPs	Start Pos	Start SNP	End Pos	End SNP
DEFB125	6	16749	rs6086616	24962	rs6111385
DEFB126	2	72302	rs6038528	73121	rs1342137
DEFB127	1	87456	rs12624954	87456	rs12624954
DEFB128	1	117995	rs6040343	117995	rs6040343
DEFB129	1	158061	rs13045643	158061	rs13045643
C20orf96	9	200165	rs513522	217703	rs11087339
ZCCHC3	0	226204	NA	228961	NA
SOX12	1	257692	rs6050276	257692	rs6050276
TRIB3	3	312747	rs7265169	325226	rs6115830
RBCK1	8	337881	rs4815585	352916	rs3787563
TBC1D20	1	373097	rs6116005	373097	rs6116005
CSNK2A1	7	416001	rs6116240	467697	rs6107514
TCF15	2	534955	rs282162	536089	rs282163
SRXN1	4	576890	rs6053666	580959	rs6085283
SCRT2	5	591677	rs6116997	600097	rs753490
C20orf54	14	688919	rs2295431	696974	rs1884637
FAM110A	1	774513	rs2282051	774513	rs2282051
ANGPT4	36	802251	rs12625616	844299	rs502522
RSPO4	19	888206	rs516714	926720	rs1154757
PSMF1	10	1058772	rs2284374	1095410	rs3087751
C20orf46	3	1110370	rs8119636	1111179	rs6040359
SNPH	17	1200492	rs7273889	1237337	rs3803949
SDCBP2	19	1238699	rs7466	1255888	rs6033448
FKBP1A	10	1298709	rs6041750	1317825	rs6109740
NSFL1C	6	1371848	rs13063	1390790	rs6131536
SIRPD	8	1466100	rs10485824	1486249	rs2250055
SIRPB1	5	1493468	rs2253693	1548524	rs1535882
SIRPG	12	1558201	rs2281807	1585879	rs6034307
SIRPA	16	1825150	rs1884565	1867324	rs7274469
PDYN	9	1908226	rs910080	1920761	rs8123727
STK35	5	2035464	rs6081971	2074180	rs2185675
TGM3	16	2234191	rs214795	2269105	rs214830
TGM6	12	2315110	rs2422761	2359792	rs7269048
SNRPB	3	2391116	rs6036572	2399451	rs6049288
ZNF343	13	2410905	rs1543385	2437531	rs6138281
TMC2	26	2465377	rs6036860	2568185	rs4813578
NOL5A	1	2583212	rs2273137	2583212	rs2273137
IDH3B	3	2587570	rs6037255	2590376	rs6115381
CPXM1	2	2723332	rs127078	2728151	rs215543
C20orf141	0	2743657	NA	2744475	NA
FAM113A	1	2765484	rs3746689	2765484	rs3746689
VPS16	10	2769601	rs2297047	2794427	rs555414

Continued on Next Page…

Locus	No. SNPs	Start Pos	Start SNP	End Pos	End SNP
PTPRA	12	2813976	rs7262267	2965113	rs566570
MRPS26	0	2974675	NA	2976895	NA
OXT	0	3000266	NA	3001162	NA
AVP	0	3011203	NA	3013370	NA
UBOX5	3	3038124	rs913555	3061818	rs857250
FASTKD5	5	3067555	rs857252	3088304	rs4815570
C20orf116	6	3121666	rs6084288	3132571	rs11697114
ITPA	9	3141508	rs6084305	3150189	rs6139036
SLC4A11	4	3158692	rs6139039	3164203	rs6051669
ATRN	22	3419971	rs238722	3578249	rs432647
GFRA4	1	3591653	rs6084432	3591653	rs6084432
ADAM33	8	3597431	rs677044	3610142	rs554743
SIGLEC1	11	3615825	rs1046919	3632960	rs735294
HSPA12B	5	3664457	rs509764	3681487	rs3088007
C20orf27	3	3683436	rs2295342	3692621	rs6139201
C20orf28	0	3706152	NA	3710061	NA
CENPB	0	3712500	NA	3715337	NA
CDC25B	1	3732110	rs1056720	3732110	rs1056720
C20orf29	2	3751428	rs6037668	3751446	rs6052095
PANK2	3	3818932	rs6084506	3844742	rs6052170
RNF24	5	3863885	rs241606	3890178	rs17287269
SMOX	10	4080664	rs1741296	4115234	rs6139351
ADRA1D	8	4156895	rs835880	4173573	rs6052456
PRNP	2	4617534	rs6116471	4628251	rs1799990
PRND	9	4650964	rs6076722	4656979	rs12625115
PRNT	7	4662227	rs1040872	4667449	rs2422932
RASSF2	26	4710577	rs3746673	4743703	rs1885305
LOC643305	7	4735677	rs6084897	4747322	rs2423045
LOC728286	1	4748521	rs1885308	4748521	rs1885308
SLC23A2	28	4782338	rs1131382	4928952	rs13042903
C20orf30	2	5029937	rs6053111	5034939	rs6116651
PCNA	1	5047516	rs17349	5047516	rs17349
CDS2	8	5057512	rs2083918	5102912	rs1040758
PROKR2	5	5231256	rs3746682	5240086	rs6516015
C20orf196	36	5679803	rs6116934	5791812	rs1699233
CHGB	6	5840853	rs2300426	5853779	rs2821
MCM8	5	5881108	rs236110	5918438	rs6053815
CRLS1	5	5936364	rs7265464	5962954	rs6139898
C20orf75	3	5973585	rs6085358	5982532	rs1884642
FERMT1	21	6004665	rs6139909	6050996	rs2144937
BMP2	3	6699316	rs7270163	6707115	rs235768
HAO1	11	7821112	rs2423322	7868432	rs2235237
TXNDC13	6	7910072	rs8764	7940324	rs4142416
PLCB1	144	8062704	rs6118083	8812177	rs1047381
LOC728357	50	8357376	rs1775201	8490269	rs13040221
PLCB4	39	9030318	rs16995546	9388840	rs2072954

Continued on Next Page…

Locus	No. SNPs	Start Pos	Start SNP	End Pos	End SNP
C20orf103	11	9443287	rs2232256	9450080	rs6140956
PAK7	83	9468142	rs12329540	9764860	rs6056921
ANKRD5	8	9971442	rs641648	9984434	rs6087120
SNAP25	20	10156748	rs3787303	10234313	rs4813925
MKKS	5	10340595	rs221668	10358968	rs6039924
C20orf94	16	10368254	rs13037906	10546076	rs1232602
JAG1	15	10566574	rs8708	10598712	rs7271215
BTBD3	5	11821317	rs2795022	11850598	rs2294967
SPTLC3	15	12937901	rs3761896	12977790	rs6078899
TASP1	20	13330443	rs6074606	13549523	rs6042256
ESF1	13	13643464	rs6079145	13709657	rs3789336
C20orf7	4	13718745	rs6074640	13739991	rs6074643
MACROD2	5	13933560	rs6042474	13981629	rs6079273
FLRT3	2	14260185	rs6110247	14265390	rs204605
C20orf23	82	16205484	rs6111006	16501701	rs6135800
SNRPB2	7	16658930	rs2073054	16666392	rs6044279
OTOR	5	16677138	rs6135876	16680004	rs12479958
PCSK2	76	17156504	rs6136033	17411917	rs6044842
BFSP1	23	17422690	rs6105762	17440755	rs1559956
DSTN	2	17504100	rs6034857	17509041	rs4814626
RRBP1	14	17544219	rs12479820	17600572	rs3790307
BANF2	16	17629255	rs911352	17659367	rs6034890
SNX5	7	17873870	rs1884709	17895337	rs17802664
C20orf72	2	17901571	rs8121408	17903153	rs6105852
OVOL2	19	17953600	rs2281543	17983837	rs6045166
CSRP2BP	13	18067407	rs6034985	18116353	rs8517
ZNF133	11	18225377	rs4814716	18243259	rs17805694
C20orf12	17	18316350	rs2076642	18395096	rs12625681
POLR3F	3	18406060	rs6081157	18410492	rs4813332
RBBP9	4	18421042	rs6075348	18422566	rs17734238
SEC23B	7	18456925	rs6075358	18488266	rs6075365
DTD1	9	18523485	rs6045492	18683635	rs4814787
C20orf79	1	18742714	rs1053834	18742714	rs1053834
SLC24A3	95	19143319	rs6112257	19649174	rs873328
RIN2	47	19818852	rs1056293	19930547	rs14344
NAT5	2	19948786	rs6136913	19955325	rs200177
CRNKL1	4	19974427	rs200185	19983030	rs6081849
C20orf26	66	19985794	rs6046580	20272341	rs6046805
hCG_2019139	4	20006992	rs6136938	20008745	rs6081869
INSM1	0	20296765	NA	20299590	NA
C20orf74	11	20324818	rs11087321	20582687	rs6112966
C20orf19	5	21063991	rs6047267	21135315	rs3790141
XRN2	4	21249477	rs6047377	21283113	rs2025811
NKX2-2	0	21439664	NA	21442664	NA
PAX1	0	21634297	NA	21644620	NA
FOXA2	0	22509823	NA	22513101	NA

Continued on Next Page…

Locus	No. SNPs	Start Pos	Start SNP	End Pos	End SNP
SSTR4	1	22965082	rs2567608	22965082	rs2567608
THBD	1	22975413	rs3176123	22975413	rs3176123
CD93	4	23010927	rs2749812	23013342	rs3746732
NXT1	1	23280297	rs6132598	23280297	rs6132598
GZF1	4	23293844	rs6048760	23299889	rs1419008
NAPB	5	23303423	rs8615	23346738	rs1016628
CSTL1	1	23372886	rs6083127	23372886	rs6083127
CST11	1	23380891	rs2249239	23380891	rs2249239
CST8	2	23422079	rs2236085	23422808	rs2224219
CST9L	1	23494639	rs2295564	23494639	rs2295564
CST9	2	23532116	rs726217	23534184	rs2983639
CST3	0	23562294	NA	23566574	NA
CST1	4	23677496	rs4465840	23679426	rs13045477
CST2	1	23753520	rs6114319	23753520	rs6114319
CST5	3	23806428	rs2285059	23807753	rs2071443
GGTLA4	0	23913687	NA	23917416	NA
C20orf39	33	24400407	rs2892183	24592927	rs735186
CST7	4	24878085	rs227653	24888521	rs1056036
C20orf3	5	24892166	rs6707	24921005	rs8184053
ACSS1	11	24935787	rs6132784	24982480	rs6050281
VSX1	3	25005698	rs6083734	25009308	rs4815368
ENTPD6	9	25129074	rs6050427	25154654	rs1044573
PYGB	5	25180604	rs3787080	25212910	rs2257712
ABHD12	4	25230967	rs746748	25308826	rs2500405
GINS1	6	25348019	rs6115177	25373808	rs6138569
NANP	3	25543123	rs958075	25548835	rs6138616
ZNF337	2	25617052	rs6138639	25620341	rs6050790
C20orf191	2	26033338	rs2697515	26036528	rs6138799
DEFB115	2	29309964	rs6060436	29310063	rs1474945
DEFB116	1	29359337	rs5001275	29359337	rs5001275
DEFB118	1	29424119	rs6057649	29424119	rs6057649
DEFB119	3	29429936	rs2223527	29436606	rs6058963
DEFB121	0	29456361	NA	29457651	NA
DEFB123	3	29493635	rs6120282	29501444	rs2273468
DEFB124	0	29516970	NA	29524477	NA
REM1	1	29535505	rs2233834	29535505	rs2233834
HM13	5	29597479	rs4911156	29614419	rs6060007
ID1	0	29656753	NA	29657974	NA
COX4I2	2	29695298	rs6060446	29696113	rs6120970
BCL2L1	2	29725820	rs6060627	29751155	rs1994250
TPX2	5	29794708	rs4453755	29848853	rs6058463
MYLK2	1	29872241	rs4911532	29872241	rs4911532
FKHL18	2	29896800	rs6089096	29897013	rs6121246
DUSP15	2	29915505	rs6087786	29916695	rs2377318
TTLL9	14	29923100	rs8120234	29990726	rs6061043
PDRG1	2	29997544	rs8122147	29997610	rs6089127

Continued on Next Page…

Locus	No. SNPs	Start Pos	Start SNP	End Pos	End SNP
XKR7	8	30022208	rs8120417	30049685	rs714605
C20orf160	1	30071707	rs6061104	30071707	rs6061104
HCK	4	30109620	rs6058522	30142213	rs17259459
TM9SF4	3	30186585	rs17266770	30209909	rs6061195
PLAGL2	3	30245774	rs6061216	30254839	rs3787371
POFUT1	3	30262329	rs6141636	30279411	rs6141641
KIF3B	4	30350567	rs4911090	30386060	rs13111
ASXL1	4	30436835	rs4911226	30482685	rs2295765
C20orf112	4	30498790	rs1737887	30512043	rs1555133
COMMD7	3	30781733	rs6141792	30793776	rs4911252
DNMT3B	4	30824044	rs2424908	30860197	rs2424932
MAPRE1	3	30893150	rs402951	30894297	rs7271735
SPAG4L	3	31049963	rs4911280	31053728	rs6059010
BPIL1	3	31063878	rs6059016	31071911	rs6057703
BPIL3	5	31083583	rs2070313	31094632	rs411001
C20orf185	6	31110189	rs6141871	31117805	rs743176
C20orf186	6	31135260	rs4339026	31151921	rs11696307
C20orf70	0	31219621	NA	31232884	NA
C20orf71	5	31270249	rs17305657	31278009	rs1570033
PLUNC	1	31291926	rs6059187	31291926	rs6059187
C20orf114	8	31337482	rs3746393	31361215	rs1999663
CDK5RAP1	4	31419715	rs6120241	31433319	rs291705
SNTA1	2	31464953	rs657040	31478697	rs6087487
CBFA2T2	2	31633261	rs6120309	31676351	rs3803939
NECAB3	2	31711824	rs3746460	31711942	rs17124895
C20orf144	0	31713781	NA	31715381	NA
C20orf134	0	31717965	NA	31719991	NA
E2F1	1	31729174	rs2071056	31729174	rs2071056
PXMP4	3	31759954	rs2377925	31768314	rs1074683
ZNF341	7	31796842	rs7274811	31842393	rs2747565
CHMP4B	5	31878750	rs736953	31890503	rs6088316
RALY	6	32051756	rs2284378	32087079	rs4911399
EIF2S2	3	32145986	rs11700255	32161200	rs6142100
ASIP	1	32315943	rs819162	32315943	rs819162
AHCY	2	32335211	rs864702	32341427	rs1205357
ITCH	4	32415786	rs12624640	32496576	rs6579167
DYNLRB1	3	32569558	rs6059893	32577862	rs8116198
MAP1LC3A	2	32603352	rs6059909	32609065	rs4911430
PIGU	7	32628465	rs6059926	32702642	rs6087613
TP53INP2	1	32758014	rs6060001	32758014	rs6060001
NCOA6	6	32783716	rs2295352	32875532	rs6088619
GGTL3	4	32898822	rs17092148	32916261	rs17122844
ACSS2	2	32934175	rs6088638	32978126	rs6088650
GSS	6	32985715	rs6087653	33006266	rs6088659
MYH7B	3	33028830	rs6120778	33050859	rs3746435
TRPC4AP	6	33056382	rs17092209	33113591	rs6060194

Continued on Next Page. . .

Locus	No. SNPs	Start Pos	Start SNP	End Pos	End SNP
EDEM2	8	33167268	rs3746429	33198566	rs945959
PROCR	1	33227612	rs2069952	33227612	rs2069952
MMP24	8	33287248	rs2425020	33327047	rs6060341
EIF6	3	33330662	rs2297789	33335075	rs2425046
FAM83C	1	33342892	rs2425050	33342892	rs2425050
UQCC	4	33373166	rs6060370	33456505	rs1204660
GDF5	2	33487376	rs224333	33489170	rs143384
CEP250	3	33531574	rs10439606	33560767	rs2236164
ERGIC3	2	33599043	rs224415	33605701	rs17092784
SPAG4	2	33671311	rs761827	33671930	rs2425067
CPNE1	1	33684569	rs6060524	33684569	rs6060524
RBM12	2	33708793	rs11696527	33712305	rs6058293
NFS1	2	33730297	rs6121026	33734506	rs6060558
C20orf52	1	33751664	rs6060567	33751664	rs6060567
RBM39	4	33770388	rs2425094	33787512	rs2425118
PHF20	6	33830691	rs6058322	33923893	rs6058339
SCAND1	0	34004960	NA	34005842	NA
C20orf152	3	34020418	rs6060729	34078777	rs2590962
EPB41L1	5	34175724	rs6141600	34238965	rs2247688
C20orf4	1	34293282	rs6058435	34293282	rs6058435
DLGAP4	10	34430715	rs4530425	34551414	rs127416
MYL9	1	34605971	rs220076	34605971	rs220076
TGIF2	3	34640513	rs2234085	34654572	rs6016004
C20orf24	1	34670739	rs555394	34670739	rs555394
SLA2	2	34685376	rs4812383	34706192	rs221310
NDRG3	2	34732082	rs221314	34786132	rs6072018
C20orf172	0	34813862	NA	34835563	NA
C20orf117	8	34841054	rs11697056	34920573	rs1146261
C20orf118	3	34940956	rs3748459	34941149	rs3748462
SAMHD1	10	34952889	rs6029941	35013095	rs6030389
RBL1	4	35073192	rs1124692	35156244	rs1744765
C20orf132	6	35163364	rs1780705	35216908	rs1615246
RPN2	5	35245940	rs16986470	35276337	rs11700106
GHRH	0	35312906	NA	35318706	NA
MANBAL	4	35358710	rs6074018	35378652	rs1043415
SRC	12	35410985	rs6017944	35455953	rs6018257
BLCAP	2	35581911	rs3795148	35587876	rs12481150
NNAT	0	35583021	NA	35585502	NA
CTNNBL1	7	35773050	rs6067377	35930329	rs4811247
VSTM2L	4	35996958	rs6096989	36005342	rs6021982
KIAA0406	8	36047192	rs6013590	36095121	rs6022515
C20orf77	6	36115324	rs17196689	36145348	rs1569907
TGM2	4	36205544	rs11696730	36226659	rs7275079
BPI	12	36366074	rs1341023	36396639	rs2154374
LBP	4	36411924	rs1609800	36435348	rs1739640
KIAA1219	5	36541160	rs1303588	36635587	rs1115600

Continued on Next Page…

Locus	No. SNPs	Start Pos	Start SNP	End Pos	End SNP
SLC32A1	0	36786519	NA	36791429	NA
ACTR5	3	36819717	rs2255210	36832902	rs6128947
PPP1R16B	15	36872238	rs6124079	36984349	rs10392
FAM83D	2	36990599	rs16987679	36996766	rs725322
DHX35	3	37034657	rs16987712	37063430	rs7270784
MAFB	0	38747929	NA	38751290	NA
TOP1	6	39115097	rs8122094	39179817	rs6129760
PLCG1	6	39203389	rs3795131	39234124	rs2235360
ZHX3	8	39247143	rs2664537	39354560	rs6124336
LPIN3	6	39403608	rs4812495	39421666	rs6065338
EMILIN3	1	39423140	rs6072352	39423140	rs6072352
CHD6	14	39482513	rs6029672	39671224	rs2425463
PTPRT	277	40135485	rs3746539	41240275	rs2425614
SFRS6	1	41524771	rs12264	41524771	rs12264
L3MBTL	5	41580071	rs6103362	41597182	rs2071968
SGK2	11	41621277	rs3752558	41646027	rs4560183
IFT52	4	41664058	rs6030978	41699064	rs6017129
FAM112A	0	41788289	NA	41788753	NA
TOX2	33	41981244	rs6031225	42130063	rs1555122
JPH2	33	42174113	rs1055716	42246601	rs6065708
C20orf111	5	42258901	rs9875	42272126	rs2143607
GDAP1L1	4	42323921	rs6031485	42337911	rs4810417
R3HDML	6	42401240	rs11907772	42407349	rs11697359
HNF4A	17	42463849	rs2071197	42492851	rs6130615
C20orf121	1	42547344	rs6031627	42547344	rs6031627
SERINC3	2	42567299	rs6017355	42574490	rs2425641
PKIG	7	42595885	rs3092796	42658880	rs244099
ADA	12	42684136	rs244078	42707830	rs2299687
WISP2	3	42780705	rs6130677	42788069	rs753740
KCNK15	1	42811284	rs4810439	42811284	rs4810439
RIMS4	4	42817246	rs6031782	42869851	rs6031807
YWHAB	4	42959551	rs2425672	42968515	rs6876
TOMM34	1	43011058	rs1884440	43011058	rs1884440
STK4	9	43033365	rs17322289	43131586	rs2267862
KCNS1	3	43154907	rs6124684	43158293	rs6017486
WFDC5	3	43172533	rs17422688	43174937	rs734685
WFDC12	1	43185681	rs6104018	43185681	rs6104018
PI3	3	43237139	rs1983649	43237936	rs2664581
SEMG2	3	43283557	rs2233895	43285657	rs6104069
SLPI	2	43315044	rs6032073	43316208	rs6130789
MATN4	3	43363073	rs2076023	43367108	rs2281018
RBPJL	3	43369569	rs2743312	43378372	rs2741500
SDC4	8	43392264	rs2070639	43410405	rs4458268
DBNDD2	4	43426953	rs2743423	43469863	rs2247619
C20orf10	2	43438045	rs760642	43439350	rs2231616
PIGT	4	43479294	rs1028306	43487763	rs707577

Continued on Next Page…

Locus	No. SNPs	Start Pos	Start SNP	End Pos	End SNP
WFDC2	0	43531808	NA	43543585	NA
WFDC6	2	43597505	rs6094159	43599001	rs6032276
SPINLW1	3	43604126	rs11594	43607837	rs765156
WFDC8	3	43616027	rs7268787	43635293	rs7264976
WFDC9	3	43670401	rs1487320	43681107	rs1487328
WFDC10A	4	43685294	rs6073822	43692517	rs1487324
WFDC11	1	43729225	rs1487318	43729225	rs1487318
WFDC13	5	43759712	rs6104293	43766466	rs980984
WFDC3	3	43838054	rs6124741	43846502	rs6073912
DNTTIP1	3	43857250	rs6104350	43861619	rs11699807
UBE2C	0	43874662	NA	43879001	NA
TNNC2	2	43885308	rs8860	43886104	rs4629
SNX21	2	43902338	rs197669	43904083	rs3848713
ACOT8	2	43913733	rs6130946	43916632	rs6130947
ZSWIM3	2	43921902	rs3761156	43934865	rs6130959
ZSWIM1	1	43944221	rs4812974	43944221	rs4812974
C20orf165	0	43948538	NA	43949645	NA
NEURL2	1	43952250	rs3817731	43952250	rs3817731
CTSA	2	43955412	rs742034	43960190	rs7270170
PLTP	5	43964245	rs553359	43974099	rs2294213
FLJ40606	1	43998089	rs3848715	43998089	rs3848715
C20orf67	1	44009909	rs7679	44009909	rs7679
ZNF335	7	44019430	rs3827066	44029952	rs3848719
MMP9	3	44072918	rs3918253	44075138	rs3787268
SLC12A5	7	44097778	rs3746515	44116556	rs6032638
NCOA5	8	44124757	rs1537028	44135574	rs16991058
CD40	6	44180389	rs1883832	44190814	rs3765459
CDH22	10	44241393	rs6131030	44312334	rs2425804
SLC35C2	3	44416924	rs12480667	44421461	rs2771285
ELMO2	2	44446096	rs3848681	44460771	rs6065979
ZNF334	2	44574807	rs366860	44575240	rs847052
SLC13A3	35	44620912	rs10218	44711576	rs1004571
TP53RK	0	44746412	NA	44751683	NA
SLC2A10	3	44776644	rs3092439	44794862	rs3091904
EYA2	57	44965529	rs6124883	45230067	rs2903940
ZMYND8	18	45274459	rs910187	45379594	rs1013715
NCOA3	11	45586555	rs11700063	45709750	rs6066426
SULF2	69	45719646	rs445219	45846679	rs6122615

For each gene assayed, the corresponding No. of SNPs, position of the first SNP‡ and its dbSNP identifier, and the position of the last SNP‡ and dbSNP identifier are given.
‡ *Where applicable i.e. for loci with available tag SNPs.*
